# Expecting parents’ use of digital sources in preparation for parenthood in a digitalised society – a systematic review

**DOI:** 10.1177/20552076221090335

**Published:** 2022-04-14

**Authors:** Caroline Bäckström, Kristina Carlén, Viveca Larsson, Lena Birgitta Mårtensson, Stina Thorstensson, Marina Berglund, Therese Larsson, Björn Bouwmeester, Marie Wilhsson, Margaretha Larsson

**Affiliations:** 1 School of Health Sciences, 101081 University of Skövde, Sweden; 2 School of Nursing, Midwifery and Social Work, Faculty of Health and Rehabilitation Sciences, The University of Queensland, Australia

**Keywords:** Pregnancy, digitalisation, antenatal, childbirth, mother, father

## Abstract

**Background:**

In today's society, people are experiencing the rapid development of digitalisation. Expecting parents may have difficulties evaluating the information online; they are not always sure which sources of information are trustworthy, and this exacerbates their feelings of anxiety. More research is needed to broaden the knowledge about how their use of digital sources may influence their health.

**Question:**

The focus of this study was to explore expecting parents’ use of digital sources and how this influences their health during pregnancy.

**Methods:**

A systematic review covered the thematic analysis of 39 articles.

**Findings:**

The analysis resulted in the following theme: *The digitalised society involves both opportunities and challenges, and expecting parents express a need for a variety of digital sources to improve their health,* and sub-themes: *Digital sources could promote parents’ health and well-being in a digitalised society*; *Consuming digital health information facilitates understanding, different feelings and social connections*; and *A variety of digital sources may facilitate parental identification and adaption to parenthood.*

**Conclusion:**

Different digital sources in our digitalised society mean access to information and opportunities to extend social connections for expecting parents. This can promote their ability to understand and adapt to parenthood, as well as to improve their health and well-being and make the parental transition. However, professional support during face-to-face consultations cannot always be exchanged to digital sources. It is important to base digital sources devoted to expecting parents and digitalisation overall on multi-sectorial collaborations and coordination between different organisations and the digital sources they provide.

## Background

Dahlberg and Segesten^1^ highlight that health is essential for the understanding of caring; hence, the goal of caring is health. Healey-Ogden and Austin^2^ claim that the three words *health*, *well-being* and *wellness* are used interchangeably, and these words are commonly employed in the caring literature. Dahlberg and Segesten^1^ purport that health is related to human existence and the experience of well-being, and they define health as ‘well-being’ together with experiences of ‘being able to’. Similarly, Healey-Ogden and Austin^2^ find well-being to be inherent in life, and therefore, a lived experience. In this study, the phenomenon of health is highlighted with an emphasis on expecting parents’ health during pregnancy in a digitalised society, including various aspects of health such as mental, psychological, physical and social.

The technical advancement in society is a ground for new, rapid digital development. Globally, people are experiencing the rapid integration of digital technology, which affects both their personal and professional lives.^3^ Different concepts are used to describe the changes that are ongoing in society regarding digital technology. According to Savić,^4^ there are differences between the concepts of *digitisation, digitalisation* and *digital transformation.* While *digitisation* refers to data conversion as a change from analogue to digital format, *digitalisation* refers to information processing and the creation of completely digital work processes. In contrast, *digital transformation* is described as representing an umbrella perspective covering both digitisation and digitalisation, which are assumed to be smaller parts in the big picture of a society's or organisation's digital transformation. Previous research has claimed the meaning of the design of digital technology to promote wellness and relationships among families,^5^ and a systematic review expanded the understanding of designing effective systems for parent-child support including reciprocity norms of the family, transparency, accessibility and enjoyable usage, to mention a few promoting factors.^6^ However, more research is needed to broaden the knowledge of digital sources for expecting parents.

The transition to parenthood is one of the most profound life changes for human beings,^7,8^ and it includes changes regarding personal identity.^9^ This transition is influenced by the individual's level of knowledge, skills and expectations.^10^ Unrealistic expectations and feelings of being unprepared inhibit the transition, whereas feelings of being prepared facilitate the transition to parenthood.^7^ Expecting parents are likely to search for health information online, and the Internet plays an important role in supporting and providing them with information.^11^ This information-seeking behaviour has been described as a ‘holistic learning process to seek meaning’, and it affects the decision-making process regarding pregnancy.^12^ Most expecting mothers have access to the Internet and use it to retrieve information about pregnancy, childbirth and the expected child.^13^ Expecting first-time mothers often use applications related to pregnancy, birth and/or child care,^14^ and expecting mothers’ decision-making processes involve seeking, collecting and assessing information from healthcare practitioners. They also search for other mothers’ experiences and for research that has been published online before making decisions based on their perceptions of safe and trustworthy information.^15^ Health literacy could be discussed from the perspectives of, for example, clinical practice in relation to risk, or as a personal means related to participation in society as within health promotion.^16,17^ Expecting mothers with low health literacy have more personal barriers to information seeking, such as not knowing how to take care of themselves during pregnancy and not knowing how to use the Internet.^18^ The World Health Organization^19^ stipulates that, to empower people's health literacy, improving access to health information and the capacity to use it effectively is paramount. *Health literacy* may also explore the cognitive and social skills that determine the motivation and ability of an individual to gain access to, understand and use information in ways that promote and maintain good health.^20^ Health literacy could be described by three dimensions of functional health literacy (reading, writing, etc.), interactive health literacy (e.g. cognitive and social skills), and critical health literacy (such as ability for analysis, and control of information),^21^ and health literacy can include media use and digital communication.^17^ Low health literacy is associated with poorer health-related knowledge and comprehension, as well as negative health outcomes associated with a limited ability to interpret medical labels and health messages or use healthcare services.^20^

Expecting parents are usually cared for within antenatal care. Internationally, the healthcare professionals responsible for antenatal care vary.^22^ It has been revealed that expecting first-time mothers tend to use the Internet to control the information provided by midwives within antenatal care.^23,24^ A systematic review described that midwives’ in antenatal care generally represent ambivalent views towards the use of digital technology, they acknowledge both benefits (i.e. modern antenatal care with opportunities for expecting mothers to make informed decisions), limitations and risks (i.e. conveyed information and negative impacts on the relationship between the midwife and the expecting mother) with technology.^25^ Instead of disregarding the use of the Internet as a source of information during pregnancy, such healthcare professionals as midwives should keep up to date with online information and direct expecting parents to high-quality sites.^11^

It is essential that expecting parents feel able to maintain their health and well-being during the transition to parenthood in a society characterised by rapid digitalisation. During the last decade, there has been an increase in the number of expecting parents who use digital sources, such as the Internet, as a primary source of health information. Health literacy skills can be related to the individual's knowledge and expectations, which can affect expecting parents’ health and transition to parenthood. Research on the digitalised society seems to provide varying results about expecting parents’ use of digital sources and how information and the presence of digital sources affect their preparation for parenthood, as well as their health, during pregnancy. Therefore, the aim of this study was to explore expecting parents’ use of digital sources and how this use influences their health during pregnancy in the digitalised society.

In this study, the term *digital source* is used as an umbrella term for different digital sources, such as mobile applications, the Internet or online forums. When referring to specific sources, these are specified in the text.

## Methods

A systematic review was considered to be an appropriate method to explore expecting parents’ use of digital sources and how this use influences their health during pregnancy in the digitalised society. We aimed to elucidate the full range of literature available in three databases covering health-related research: PubMed, CINAHL and Scopus. Therefore, all articles that dealt with the relation between parents’ health and digitalisation were examined, regardless of study design. The systematic review was carried out according to the Preferred Reporting Items for Systematic Reviews and Meta-Analysis (PRISMA) statement,^26^ and an a priori protocol was designed outlining the aim and procedure for the review.

### Search strategy

A comprehensive and systematic search was conducted through a series of electronic searches in PubMed, CINAHL and Scopus in November 2019.

#### Search string

The search string was built by combining key terms related to the aim of the study as follows: TITLE-ABS-KEY((digit* OR computer* OR smartphone* OR online* OR tablet* OR surfpad* OR "surf pad" OR "surf pads" OR "social media" OR "mobile application" OR "mobile applications" OR internet* OR web) AND (pregnan* OR labour* OR labor* OR birth* OR childbirth* OR "child birth" OR "child-birth") AND ((parent* OR father* OR mother*) OR (family* OR familie*)) AND (health* OR "well-being" OR "well being" OR wellbeing OR "quality of life") AND (soci* OR psych* OR mental*)). Appropriate standardised vocabulary and truncation were used for each database.

#### Process of data collection

A total of 3506 publications were found after the removal of duplicates. Assessment of titles and abstracts was divided into three groups of researchers (ML and MW; KC and VL; CB and ST) and assessed independently by the authors in pairs. The assessment was based on the following inclusion criteria: (1) articles with results that revealed how digitalisation related to parents’ health during pregnancy and (2) articles published between 2007 and 2019 (November). Studies were excluded from analysis if they met one of the following criteria: (1) they focussed on pre-gestation or the postpartum period, (2) they were written in a language other than English or (3) they focussed on the views of healthcare professionals.

**Figure 1.**
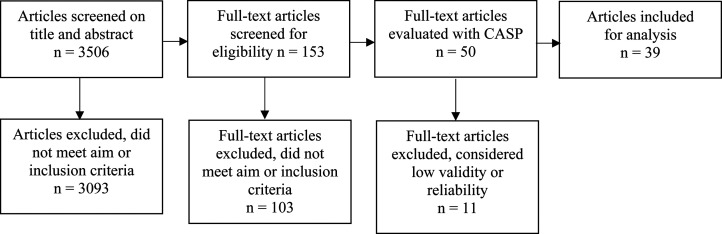
Overview of study selection.

This process ended with the exclusion of 3093 articles. Assessment of the full text of the remaining articles (*n* = 413) was divided between all authors, which corresponds to approximately 45 articles per author. This process ended in the inclusion of 153 articles that were assessed and read between teams of three groups of authors (ML and MW; KC, CB and LMB; VL and TL). Furthermore, 103 articles were excluded because they either did not meet the aim of the study or did not fulfil the inclusion criteria. The Critical Appraisal Skills Programme (CASP) Appraisal Checklists were used for evaluating the validity and reliability (e.g. the CASP used was adjusted for the specific article evaluated, for example, when evaluating a qualitative article, the specific CASP for qualitative articles was used).^27^ CASP was used evaluating 50 articles, and 11 articles were excluded considered low validity or reliability. This led to a final sample of 39 articles to be included for data extraction. The review process followed the PRISMA guidelines.^26^ For an overview of study selection, see Figure 1.

### Data extraction and analysis

To analyse the data, an iterative process was conducted for the completion of the text of the included studies. A thematic analysis was used for identifying, analysing and reporting patterns (themes) within data according to the guidelines in the step-by-step guide described by Braun and Clarke.^28^ When conducting thematic analysis, the process of analysis starts when the analyst begins to identify patterns of meaning, answering the study aim. For this study, in the first phase of the analysis – *familiarising yourself with your data* – the researchers read and re-read the included studies, and notes were taken for initial ideas about patterns of meaning. In the second phase, *generating initial codes*, the production of the initial codes was carried out. In total, 26 codes were identified as a feature of the data and referred to elements that were assessed to be significant regarding the phenomenon under study were created. In the third phase of analysis, *searching for themes*, the codes were put together into potential themes, giving a coherent picture of all data relevant to each potential theme. In the fourth phase, *reviewing themes*, the themes were controlled in relation to the coded extracts and the entire dataset to be able to generate a thematic map of the analysis. Three themes emerged. Thereafter, an ongoing analysis was carried out to identify the specifics of each theme, as well as an overall understanding that the analysis generated in the presented overall theme. This resulted in definitions and names for each theme, with two or three belonging codes, in the fifth phase of analysis—*defining and naming themes.* Finally, the sixth phase of analysis – *producing the report* – included writing the report and conducting an analysis that ‘went back and forth’ between writing and controlling themes with the data. Some of the researchers participated in the different phases of analysis (CB; KC; VL; BS; MW); all the researchers agreed on a consensus and contributed with their different experiences and professions during the last phase of analysis. The current study represents a multi-professional (i.e. public health, midwives, and nurses with bachelor and various master degrees; district, anaesthetics, child and psychiatric) group of researchers with various degrees (MSc, PhD-student, PhD, Assistant Professor and Professor) and experiences of systematic reviews and thematic analysis.

In this systematic review, different terms for expecting parents are used, such as *expecting mother/father/co-mother and/or parent*. The term *mother* is used when referring to results which relate to the pregnant woman, the term *father* refers to results concerning expecting fathers, and *co*-*mother* refers to the unpregnant woman within an expecting lesbian parental couple. When referring to results that deal with both parents, the term *parent* is used unless the parent's specific role is stated within the findings of the articles included in the analysis.

## Results

A clarification of the articles included in the analysis is presented in Table 1.

**Table 1.** Overview included articles.

Article ID	Authors	Study design	Participant selection	Sample size	Date	Country
1^61^	Åsenhed, Kilstam, Alehagen & Baggens	Qualitative: Explorative	Blogs from the Internet	Blogs from the Internet by 11 first-time fathers	2013	Sweden
2^30^	Zhao & Basnyat	Qualitative: Explorative	Forum messages from the Internet	17 605 forum messages from the Internet	2018	China
3^66^	Litchman, Tran, Dearden, Guo, Simonsen & Clark	Qualitative: Explorative	Blogs from the Internet	Blogs from the Internet by 125 expecting or new mothers	2019	USA
4^53^	Lupton	Qualitative: Focus group interviews	Purposive sampling	Focus group interviews with 36 expecting and new mothers	2016	Australia
5^56^	AlJaberi	Qualitative: Focus group interviews	Criterion-based sampling	Focus group interviews with 12 expecting and new mothers	2018	USA
6^54^	Vamos, Merrell, Detman, Louis & Daley	Qualitative: Focus group interviews	Purposive and convenience sampling	Focus group interviews with 17 expecting mothers	2019	USA
7^47^	Song, West, Lundy & Smith	Qualitative: In depth interviews	Snowball sampling	Interviews with 32 expecting mothers	2012	USA
8^46^	Fleming, Vandermause & Shaw	Qualitative: In depth interviews	Purposive sampling	In-depth interviews with 7 expecting first-time mothers	2014	USA
9^64^	Entsieh, Emmelin & Pettersson	Qualitative: In depth interviews	Purposive sampling	In-depth interviews with 25 expecting and new mothers	2015	Ghana
10^62^	Johnson	Qualitative: In depth interviews	Purposive sampling	In-depth interviews with 22 expecting first-time mothers	2015	Australia
11^60^	McCarthy, Choucri, Ormandy & Brettle	Qualitative: In depth interviews	Purposive sampling	In-depth interviews with 31 mothers	2017	UK
12^48^	Liechty, Coyne, Collier & Sharp	Qualitative: In depth interviews	Purposive sampling	In-depth interviews with 50 expecting mothers and 26 mothers	2018	USA
13^36^	Johnsen, Clausen, Hvidtjorn, Juhl & Hegaard	Qualitative: In depth interviews and questionnaire	Purposive sampling	In depth interviews, questionnaire and observation with 15 expecting women	2018	Denmark
14^29^	Berg, Linden, Adolfsson, Sparud Lundin & Ranerup	Qualitative: Questionnaire	Convenience sampling	Questionnaires with 81 expecting mothers	2018	Sweden
15^49^	Carolan	Qualitative: Repeated in depth interviews	Purposive sampling	Interviews with 22 first-time mothers	2007	Australia
16^57^	Edwards, Speight, Bridgman & Skinner	Qualitative: Written interactions	Purposive sampling	Written interactions from 93 expecting mothers	2016	Australia
17^63^	Kennedy, Mullaney, Reynolds, Cawley, McCartney & Turner	Quantiative: Questionnaire	Convenience sampling	Questionnaires with 110 expecting mothers	2017	Ireland
18^44^	Zlotnick, Tzilos & Raker	Quantiative: Randomized controlled trial with questionnaires	Purposive sampling	Questionnaires with 443 expecting or new mothers	2019	USA
19^51^	Hämeen-Anttila, Nordeng, Kokki, Jyrkkä, Lupattelli, Vainio & Enlund	Quantitative: Questionnaire	Convenience sampling	Questionnaires with 5090 expecting mothers	2014	Europe, America, Australia
20^50^	Lupton & Pedersen	Quantitative: Questionnaire	Purposive sampling	Questionnaires with 410 expectant and new mothers	2016	Australia
21^65^	Wallwiener, Muller, Doster, Laserer, Reck, Pauluschke-Fröhlich, Brucker, Wallwiener & Wallwiener	Quantitative: Questionnaire	Purposive sampling	Questionnaires with 220 expecting mothers	2016	Germany
22^34^	Da Costa, Zelkowitz, Letourneau, Howlett, Dennis, Russel, Grover, Lowensteyn, Chan & Khalifé	Quantitative: Questionnaire	Purposive and convenience sampling	Questionnaires with 174 expecting or new fathers	2017	Canada
23^55^	Özkan Sat & Yaman Sözbir	Quantitative: Questionnaire	Purposive sampling	Questionnaires with 230 expecting mothers	2018	Turkey
24^58^	Oscarsson, Medin, Holmström & Lendahls	Quantitative: Questionnaire	Purposive sampling	Questionnaires with 92 fathers	2018	Sweden
25^41^	Acquavita, Krummel, Talks, Cobb & McClure	Quantitative: Questionnaire	Purposive and convenience sampling	Questionnaires with 170 expecting and new mothers	2019	USA
26^52^	Song, Cramer, McRoy & May	Quantitative: Questionnaires	Convenience sampling	Questionnaires with 63 expecting mothers	2013	USA, Wisconsin
27^38^	Ledford, Womack, Rider, Seehusen, Conner, Lauters & Hodge	Quantitative: Randomized controlled trial	Purposive sampling	Questionnaires with 241 expecting mothers	2018	USA
28^39^	Kingston, Austin, Veldhuyzen van Zanten, Harvalik, Giallo, McDonald, MacQueen, Vermeyden, Lasiuk, Sword & Biringer	Quantitative: Randomized controlled trial with questionnaire	Purposive sampling	Questionnaires with 636 expecting mothers	2017	Canada, Alberta
29^40^	Larsson, Karlström, Rubertsson, Ternström, Ekdahl, Segebladh, Hildingsson	Quantitative: Randomized controlled trial with questionnaires	Purposive sampling	Questionnaires with 258 expecting mothers	2017	Sweden
30^37^	Abbasi, Mohammad-Alizadeh Charandabi & Mirghafourvand	Quantitative: Randomized controlled trial with questionnaires	Purposive sampling	Questionnaires with 153 expecting mothers	2018	Iran
31^32^	Krusche, Dymond, Murphy & Crane	Quantitative: Randomized controlled trial with questionnaires	Convenience sampling	Questionnaires with 185 expecting mothers	2018	UK
32^33^	Loughnan, Sie, Hobbs, Joubert, Smith, Haskelberg, Mahoney, Kladnitski, Holt, Milgrom, Austin, Andrews & Newby	Quantitative: Randomized controlled trial with questionnaires	Convenience sampling	Questionnaires with 409 expecting mothers	2019	Australia and New Zealand
33^31^	Yang, Jia, Sun, Ye, Zhang & Yu	Quantitative: Randomized controlled trial with questionnaires	Purposive sampling	Questionnaires with 123 expecting mothers	2019	China
34^45^	Linden, Berg, Adolfsson & Sparud-Lundin	Quantitative: Randomized controlled trial with questionnaires	Purposive sampling	Questionnaires with 174 expecting mothers	2018	Sweden
35^43^	Tzilos Wernette, Plegeue, Kahler, Sen & Zlotnick	Quantitative: Randomized controlled trial with sample testing and questionnaires	Purposive sampling	Questionnaires and sample testing with 50 expecting mothers	2018	USA
36^72^	Mackert, Guadano, Donovan & Whitten	Mixed methods with qualitative semi-structured interviews and quantiative	Purposive sampling	Qualitative interviews and quantitative questionnaires with 32 men	2015	USA
37^42^	Marshall, Moon, Mirchandani, Smith, Nichols, Zhao, Vydiswaran & Chang	Mixed methods: Explorative	Purposive sampling	Extraction of Facebook posts from 43 expecting mothers	2019	USA
38^59^	Mackert, Guadagno, Lazard, Donovan, Rochlen, Garcia, Damasio & Crook	Mixed methods: Questionnaire	Convenience sampling	Questionnaires with 962 men	2018	USA
39^35^	Dalton, Rodger, Wilmore, Humphreys, Skuse, Roberts & Clifton	Mixed methods: Questionnaire	Purposive sampling	Questionnaires with 150 expecting mothers	2018	Australia

The analysis resulted in an overall theme and three themes with associated codes, as presented in Table 2.

**Table 2.** Overview of overall theme, themes and codes.

Overall theme	Themes	Codes
The digitalised society involves both opportunities and challenges, and expecting parents express a need for a variety of digital sources to improve their health	Digital sources could promote parents’ health and well-being in a digitalised society	Programmes provided through digital sources could reduce anxiety and worry
		Programmes provided through digital sources cannot always replace face-to-face interventions
		Programmes provided through digital sources could promote expecting parents’ empowerment
	Consuming digital health information facilitates understanding, different feelings and social connections	Consuming information through digital sources facilitate feelings of knowledge and control
		Exchange of experiences induces mixed feelings and influences decision making
	A variety of digital sources may facilitate parental identification and adaption to parenthood	Various digital sources facilitate parental transition and identification processes
		Willingness to feel normal or induce unrealistic expectations
		Needs for and ability to ensure discretion

### Overall theme: The digitalised society involves both opportunities and challenges, and expecting parents express a need for a variety of digital sources to improve their health

The digitalised society necessitates access to information and possibilities to extend social connections for expecting parents using various digital sources. Access to information and extended social networks can promote expecting parents’ ability to understand and adapt to parenthood, as well as improving their health. Expecting parents want reassurance, and they require information that is in line with their unique personal needs. However, using digital sources for health issues seems to be affected by socio-economic and cultural aspects. Digital sources that extend social connections also imply being introduced to a variety of others’ experiences that can be both empowering or a cause for concern. This contradiction highlights expecting parents’ needs for professional support to guide them in selecting relevant and credible information. Sometimes, they prefer face-to-face consultations compared with digital sources and sometimes they do not, because digital sources could both be experienced as facilitating anonymity and as threatening confidentiality. Expecting parents’ use of digital sources seems to influence their health in a positive way, but it can also cause anxiety. Regardless of which emotions arise in the expecting parent when using digital sources, these emotions will affect the transition to parenthood.

### Theme 1: digital sources could promote parents’ health and well-being in a digitalised society

Expecting parents use different digital sources, such as online discussion forums or health intervention programmes introduced by professionals, to promote their health. Programmes provided through digital sources could promote parents’ empowerment and self-efficacy, thereby affecting their health. However, expecting parents sometimes prefer face-to-face interventions instead of interventions through digital sources.

#### Programmes provided through digital sources could reduce anxiety and worry

The literature shows that access to digital sources, such as mobile applications or online forums, could improve expecting parents’ health. Online parenting forums are helpful for expecting mothers to ask questions, which facilitate their feelings of calmness and abilities to stay focussed on pregnancy-related issues^29^ and may reduce feelings of depression or anxiety.^30^ Expecting mothers describe that they feel calm, relaxed and energetic, as well as becoming aware of foetal movement, when practicing mindfulness online.^31^ Online mindfulness programmes may help expecting mothers reduce their depressive or anxious symptoms, as well as to improve and maintain an accepting attitude.^31,32^ They also feel less depressed, distressed and worried about labour issues.^32^ Internet-based cognitive behavioural therapy for expecting mothers also indicates a reduction in anxiety, distress and depression.^33^ While, psychologically distressed expecting fathers seem to have a stronger need for practical strategies to improve their emotional well-being.^34^ Dalton *et al*.^35^ report that, in terms of expecting mothers’ ability to access and use mobile technology, it is important to notice women's level of technological literacy to ensure that applications are accessible, so their ability to engage with health professionals and educational applications through digital sources is strengthened. When working with strategies to reduce expecting fathers’ stress and anxiety, the language is important to overcome gender-tailored factors.^34^

#### Programmes provided through digital sources cannot always replace face-to-face interventions

Expecting parents seem to benefit from digital sources, but sometimes, they prefer face-to-face interventions. The experience of being anonymous online can make it easier to state personal problems than to sit face to face with the midwife and verbalise them.^36^ Using e-learning was more effective on expected self-efficacy than using a training booklet.^37^ In addition, expecting mothers using a mobile application interface more frequently recorded information about their pregnancy and health compared with mothers who used only a paper notebook.^38^ However, there were no reported differences in health outcomes between expecting mothers using a mobile application to replace a paper notebook guide as a patient education and engagement tool in the prenatal clinical setting.^38^ There were no differences in emotional health concerns between expecting mothers using mental health e-screening and those answering on paper.^39^ Expecting mothers with childbirth fear were more satisfied with face-to-face counselling to reduce their fear than they are with Internet-based Cognitive Behaviour Therapy.^40^ Further, engaging in their health, expecting mothers preferred face-to-face counselling over Internet-based communication or telephone, not least because they were concerned about confidentiality when using the Internet or telephone.^41^

#### Programmes provided through digital sources could promote expecting parents’ empowerment

The literature is not consistent on whether digital sources could promote empowerment in expecting parents. On the one hand, studies have shown that online parenting forums positively affect single expecting mothers’ overall well-being and self-esteem and promote their individual empowerment,^30^ body image and self-esteem.^42^ Socially disadvantaged expecting mothers’ potential to inform, educate and change behaviour is also positively influenced by online parenting forums.^35^ Further, e-learning positively affects expecting mothers’ beliefs regarding labour desirability and their childbirth self-efficacy.^37^ Focussed motivational training through online professional interventions facilitates a reduction of marijuana and alcohol use among expecting mothers.^43^ Internet-based support with motivational interviewing principles can help reduce intimate partner victimisation among expecting mothers, facilitate the mothers’ preparedness in dealing with problems and reduce their emotional and physical abuse.^44^

On the other hand, Web-based professional support has not shown any impact on self-efficacy and disease management among expecting mothers with chronical disease.^45^ However, when partaking in a digital community (i.e. electronic linkages, mobile phone technology, videos and access to provider and hospital websites), expecting mothers may be exposed to information, images^46^ or others’ negative childbirth experiences^47^ they do not want to take part in.^46^ This may arouse feelings of fear,^46^ and expecting mothers have to choose when to use social media.^48^ Sometimes, the online information, provided through the Internet or mobile applications, is described as empowering; on other occasions, however, it can terrify, trigger worries and promote feelings of anxiety or upset.^49,50^

### Theme 2: consuming digital health information facilitates understanding, different feelings and social connections

When expecting parents use digital sources to view health information, it can facilitate their understanding as a way to feel informed, strengthened and in control. Information could be provided both by professionals, who expect parents to prefer their resources when searching for credible information, and by others. An exchange of experiences between expecting parents and others could induce mixed feelings, such as arouse anxiety or feelings of recognition.

#### Consuming information through digital sources facilitates feelings of knowledge and control

Research shows differences in the use of digital sources. Hämeen-Anttila *et al*.^51^ report that 60% of expecting mothers seek information from multiple digital sources, and Song *et al*.^52^ report that low-income expecting mothers rarely use digital sources for health-related information. Expecting mothers use digital sources to obtain knowledge for motherhood,^46,49^ which could be described as self-education^46^ that facilitates comfort and reassurance.^53^ They want digital information to be immediate, regular, detailed and entertaining. Further, the information should be customised, practical, professional, reassuring and unbiased.^53^ Information provided by healthcare professionals is preferable,^49^ as they are considered credible and reliable sources who provide accessible and understandable information that is applicable in real life.^54^ Expecting mothers search for information concerning the different pregnancy trimesters,^41,53,55^ and foetus^50^ and child development.^41,55^ They want specific sociocultural information and support in line with their needs,^56^ which are related to their contemplation of pregnancy planning, conception, pregnancy loss, delivery, birth and motherhood.^57^ Sometimes, expecting mothers turn to digital sources when feeling abandoned by medical professionals,^56^ and they search for additional information, which can lead to them feeling in control over the situation.^47^

Expecting fathers consider online websites to be useful for pregnancy-related information;^58^ they want information concerning baby care, healthy diet, personal stress levels and how to improve the couple relationship with their partner after delivery.^34^ Such information has been shown to increase expecting fathers’ understanding of foetus development and fragility, as well as their understanding of the importance of a healthy diet during pregnancy.^59^ Expecting first-time fathers may be distressed by the information they receive from online websites, and they will need to address these issues with a midwife in antenatal care. In contrast, expecting fathers who already have children seldom have the same concerns.^58^ Expecting fathers want digital information to be relevant on a personal level, as well as practical and based on scientific information. They perceive information with these characteristics as credible. In addition, the norms of masculinity are essential and affect the use and sustainability of websites providing information to expecting fathers.^34^

#### Exchange of experiences induces mixed feelings and influences decision making

The literature shows that expecting parents value the possibility of expanding social connections through digital sources. A social network is essential for expecting parents to have health-related questions answered; it also enables room for emotional reactions.^36^ Both expecting mothers^53^ and fathers^58^ want connections with others in the same situation, and both mothers^53,60^ and fathers^61^ want to share experiences. Expecting mothers use social media, text messaging groups and Web-based communication sources for contacts with their family members^50^ or other social networks.^56^ Reading blogs may strengthen the relationship between the expecting mother and her mother.^55^ When expecting mothers seek advice from others (i.e. knowledge and information outside the medical profession), the exchange of knowledge has been described as practical ‘phronesis’, containing a knowledge that has to be redefined by the recipient. In cases when the advice given from digital sources is not in line with healthcare professionals’ recommendations, it can lead the expecting mother to a sense of security^62^ despite the information—especially nutritional information—, sometimes, not being evidence-based.^63^ The information provided by the Internet or mobile applications could be experienced as an overload or irrelevant,^49,50^ requiring expecting mothers to filter it.^46^ Expecting parents’ abilities to choose between conflicting pieces of information are associated with their country of origin,^51^ ethnicity and employment,^54^ and education level.^51,54^ However, another study found no associations between fathers’ health literacy and their use of mobile applications for parental support.^59^ Entsieh *et al*.^64^ suggested that mHealth applications could help expecting mothers to balance knowledge from the local community and traditional practices (i.e. generational knowledge passed on through grandmothers) and traditional birth attendants together with knowledge brought by medical professionals. Information gathered from digital sources helps expecting mothers to listen to and grasp advice and question harmful practices; it also involves fathers in the preparations for childbirth.^64^ The information expecting mothers receive from others through social media may influence their decision making regarding childbirth-related questions.^47^

### Theme 3: a variety of digital sources may facilitate parental identification and adaption to parenthood

Digital sources used by expecting parents affect their parental transition and identification processes. They reflect on themselves with others and want to be similar to other parents; they do not want to feel different. Expecting parents want to be able to choose discretion when using digital sources and not reveal their identities. They use digital sources to interact with other parents to increase their feeling of belonging.

#### Various digital sources facilitate parental transition and identification processes

Expecting parents use digital sources to prepare for parenthood and to identify themselves as parents.^46,47,49,53,62^ Information about parenthood provided through applications is essential for expecting fathers to become involved in the preparations for becoming a parent,^59,61^ and applications may help expecting mothers adapt not only to the pregnancy but also to the relationship with the expecting father.^55^ Expecting fathers’ blogs often concern aspects of fatherhood,^61^ and expecting mothers’ online networks sometimes work as arenas where they can challenge and test their new role and identity as a way to legitimise their parental identity.^62^ Online forums for single expecting mothers affirm their value and encourage a positive and confident view of themselves.^30^ Social media has been described as viewing experiences of ‘real people’; expecting mothers appreciate the opportunity to identify with other real people.^48^ One study stated that expecting mothers who have migrated feel challenged by the information and images published on social media because they have difficulties relating to it, and they experience emotional stress and a lack of social support.^56^ Another study stated that first-time expecting mothers increased their influence from the information obtained from digital sources when using digital sources frequently. However, women's age seems to affect the frequency and use of digital sources, such as younger, first-time mothers were more likely to use mobile applications and to be influenced by the information.^65^

#### Willingness of feeling normal or inducing unrealistic expectations

Expecting mothers use digital sources, such as the Internet, to confirm their perceptions and experiences of what is normal in relation to both physical and psychological experiences of pregnancy. They also search for reassurance and confirmation of their normality as pregnant women.^47^ Expecting mothers perceive that images posted on social media show unrealistic pregnancies manufactured as ‘normal pregnancies’, which do not represent the wide range of varying experiences. As a result, this could facilitate unrealistic expectations among expecting mothers, which may negatively affect their feelings of normality.^48^ For example, expecting mothers may perceive that the images published on social media show the beauty of being pregnant, and they may want to look more like the women at the images;^48^ it has been found that expecting mothers with dark skin want images that reflect female bodies with a skin colour representative of their own.^56^ Further, support received through online parenting forums has a positive impact on single mothers’ well-being because the presence and exchange of support raises group consciousness and creates a bond over a sense of belonging to an online community.^30^ In addition, expecting mothers with physical disabilities may have other information needs, and their preparation for childbirth and parenthood may differ even though they experience common pregnancy symptoms. Therefore, expecting mothers who use a wheelchair, for example, sometimes start blogs to find peers they may relate to, which offers them a sense of shared experience.^66^

#### Needs for and ability to ensure discretion

Digital sources, such as online communities, allow expecting parents to interact without revealing their identities. This discretion means that digital sources facilitate for and provide opportunities to ask sensitive questions,^58,62^ providing a type of ‘surreptitious support’.^62^ Expecting mothers use social media for discretion regarding pregnancy-related issues.^56^ This brings opportunities for them to share highly personal details with little social cost.^47^ Sometimes, expecting mothers’ cultural beliefs may raise thoughts that sharing experiences online might cast a curse and lead to misfortune. Those expecting mothers choose to use private chat rooms in groups of mothers that share their cultural beliefs.^56^ In addition, expecting mothers with physical or physiological disabilities sometimes experience that it is more comfortable to share their pregnancy online, at personal blogs, instead of doing it in person.^66^ Regarding healthcare professional–initiated e-screening, expecting mothers may feel more comfortable telling the truth about their emotional health compared with doing so using paper-based screening. E-screening was not considered impersonal; whereas face-to-face screening is perceived as a potential risk if the expecting mothers do not feel comfortable answering the screening questions.^39^ Secure online communities are well-elaborated supplements for regular maternal care through timely access to information.^60^

## Discussion

According to our results, expecting parents’ use of digital sources influence their preparation for parenthood, as well as their health during pregnancy in the digitalised society. The expecting mothers in the articles prepared themselves and were self-educated through various digital sources,^46^ and the information received influenced their decision making about pregnancy-related issues.^47^ This is in line with the information-seeking process described as a ‘holistic learning process to seek meaning’.^12^ The current results showed that the information obtained through digital sources was not always in line with healthcare professionals’ recommendations, and thus, led to a false sense of security.^46^ There was a difference in how expecting parents used digital sources; for example, low-income pregnant women rarely used the Internet for health-related information,^52^ and expecting first-time fathers could become distressed when exposed to the information online,^58^ which could be due to norms of masculinity.^34^ This could influence their ‘holistic learning process to seek meaning’^12^ and may be understood as suggesting that the expecting parents’ health literacy skills influence their relation to digitally provided information, since low health literacy can be related to individual barriers to seeking information digitally,^18^ while low health literacy is associated with poorer health-related knowledge.^21^ It seems important that professionals meeting expecting parents show interest in their health literacy level to be able to provide individual support (i.e. person-centred support).

In our findings, expecting parents are helped by using various types of digital sources (such as the Internet, mobile applications or multi-functional digital platforms, social media, online forums, personal blogs, videos or access to hospital websites) in their identification as parents, which is part of the parental transition. Sometimes, expecting parents are passive consumers of digitally provided information, and, sometimes, they are reflective.^36^ Nevertheless, they want to feel like others and to obtain information they could identify themselves with. For example, expecting mothers with dark skin want images that shows diverse pregnant bodies, not just mothers’ bodies with white skin.^56^ The expecting parents expressed the need for information in both text and images that they could identify with. Therefore, healthcare professionals should be given opportunities to develop digitally based information that could be individually adapted for the wide range of expecting parents who are active online or at least be able to give advice about digital sources with evidence-based information.

The results of our study showed that parents sometimes need to remain anonymous;^39^ they want to be able to choose discretion when using digital sources.^58,62^ Several factors were shown to facilitate expecting parents’ use of digital sources, such as their age,^67^ socio-economic status,^52^ culture^56^ and other individual prerequisites, such as disabilities.^66^ A reflection is that these factors can influence parents’ abilities to evaluate information.

Previous research has shown that digital sources may be valuable for parents from a longitudinal perspective other than pregnancy, which has been the focus for this study. For example, first-time mothers may initiate breastfeeding to a larger extent.^68^ Therefore, further research is needed on parents’ use of digital sources during the postnatal period.

In today's society, humans are experiencing the rapid integration of digital technology that affects both their personal and professional lives^3^; at the same time, expecting parents are experiencing the parental transition as one of the most drastic changes in their lives,^7^ such that they may be extra-vulnerable to the integration of digital technology. There is a constant flow of information in today's society, and the individuals within the society are affected by the information flow; they might experience difficulties in choosing whether they want to take in the information. They may experience feelings of being overloaded by information.^49^ The results of the current study showed that expecting mothers express that they have to choose to take it in (i.e. the digital information flow^48^). However, expecting parents’ abilities to choose may vary. The constant information flow demands that individuals sort themselves within it, and they are forced to relate to the information projected to them. Sometimes, the digitally projected information does not match the information provided by healthcare professionals, which could cause feelings of concern among the parents.^62^ In addition, individuals come from different backgrounds, and thus, they may be more or less equipped to decide on the value of the information. Understanding the consequences of these conditions is the core of digital health literacy. Therefore, health professionals who meet expecting parents should investigate the parents’ health literacy to be able to support them in how to use digital sources during pregnancy. For this to succeed, healthcare professionals should be aware that health literacy goes beyond health education and individual behaviour-oriented communication; it includes environmental, political and social factors that influence an individual's health.^19^ Questions like the following are worth considering: *Who uses digital sources? What factors influence such use? What effects does such use have?* Further, it is valuable to consider which individuals do not use digital sources and what it entails. The rapid development of digitalisation in global society may be generational instead of age related. Therefore, healthcare professionals should strive to strengthen expecting parents’ digital health literacy because then the parents may be able to strengthen their children's digital health literacy when it is time for them to become active consumers of the digital society.

The results of the current study contribute knowledge on expecting parents’ use of digital sources during pregnancy and its relation to their health. Such knowledge may lead to healthcare professionals’ further developed competence concerning digitalisation and digital health literacy, which could lead to their better abilities to develop and recommend digital sources for expecting parents. However, to make specific digital sources and digitalisation overall work for expecting parents and the healthcare professionals who support them, it is important to encourage multi-sectorial collaborations and coordination between different organisations and the digital sources they provide.^69^ Then, the information and support provided through digital sources may become more secure and cost-effective not only for specific individuals but also for global society overall.^69^ This is because, today, such individuals as expecting parents are being affected by the digital society without always being aware of it.

### Strengths and limitations

A first limitation is that some of the articles included in the analysis covered both the pregnancy and postpartum periods. Sometimes, it was difficult to determine whether the results in the articles related to pregnancy or the postpartum period. In cases where it was not clearly specified that the result was derived from the period of pregnancy, the result was excluded from the analysis for the current review. A strength of this review is that it covers 39 articles representing 13 countries. However, the countries represented are predominantly Western, and therefore, the exclusion of articles written in languages other than English might have hampered the transferability of the results. Yet, the results did not show any difference based on country. We did not find any differences based on publication year, except that the number of articles published increased every year. A likely explanation for this is the rapid development of digital sources and increasing digitalisation within society. Another strength of this review is that we included articles that met the study aim, regardless of the study design. In total, this review included 16 articles with qualitative methods, 19 articles with quantitative methods and 4 articles with mixed methods. From this, we can conclude that there seems to be a balance between the use of research methods for studying the relation between digitalisation and parents’ health during pregnancy. In addition, this review covers only pregnancy, and thus, it does not relate to the whole parental transition. Therefore, future studies could explore the relation between digitalisation and parents’ health during the parental transition from a longitudinal perspective. Current study has a health perspective and focusses, therefore, not particularly on the specific technologies used in the included articles (such as the Internet, mobile applications or multi-functional digital platforms, social media, online forums, personal blogs, videos or access to hospital websites). This might be argued as a limitation because the design of the digital source for parental support aligns with the effectiveness of the same. For example, a systematic review showed that when designing effective systems for parent-child support, factors such as: norms, transparency and trust, interface design, accessibility, user experience and context are valuable to consider.^6^ In addition, current study was performed before the COVID-19-pandemic which brought with restrictions that influenced on expecting parents’ feelings of social isolation,^70^ and the use of digital sources in antenatal care to reduce pregnancy-related distress and anxiety,^71^ for example. The role of the continuously developing technology, due to the COVID-19-pandemic, in regard to the health among parents is claimed to need further exploration.^6^ Therefore, we suggest future reviews to focus on parents’ use of digital sources and how this use influences their health during pregnancy in the digitalised society in the post-COVID-19-pandemic phase.

## Conclusion

The results of this study show how different digital sources within our digitalised society represent access to information and opportunities to make extended social connections for expecting parents. Such access to information can promote their ability to understand and adapt to parenthood, as well as to improve expecting parents’ health and well-being; this could facilitate the parental transition. The results contribute increased knowledge that can improve healthcare professionals’ competencies concerning digitalisation and digital health literacy among expecting parents, which could lead to their better abilities to develop and recommend digital sources for expecting parents. However, this study suggests that it is important for digital sources devoted to expecting parents and digitalisation overall to be based on multi-sectorial collaborations and coordination between different organisations and the digital sources they provide. Furthermore, this study reveals that professional support during face-to-face consultations cannot always be exchanged to digital sources. Further research is needed to explore how parents’ use of digital sources may influence their health from a longitudinal perspective.
